# Text Messaging and Home Blood Pressure Monitoring for Patients with Uncontrolled Hypertension: Proposal for a Feasibility Pilot Randomized Controlled Trial

**DOI:** 10.2196/18984

**Published:** 2021-05-14

**Authors:** Claudia L Campos, Deanna Jones, Beverly M Snively, Michael Rocco, Carolyn Pedley, Sara Atwater, Justin B Moore

**Affiliations:** 1 Department of Internal Medicine Wake Forest School of Medicine Winston-Salem, NC United States; 2 Department of Biostatistics and Data Sciences Division of Public Health Sciences Wake Forest School of Medicine Winston-Salem, NC United States; 3 Department of Family and Community Medicine Wake Forest School of Medicine Winston-Salem, NC United States; 4 Department of Implementation Science Wake Forest School of Medicine Winston-Salem, NC United States

**Keywords:** hypertension, home blood pressure monitoring, telehealth, medication adherence, SMS, health disparities

## Abstract

**Background:**

A decrease in blood pressure, even modestly (ie, 2 mmHg), lowers cardiovascular morbidity and mortality. Low patient adherence to antihypertensive medication is the most significant modifiable patient-related barrier to achieving controlled blood pressure. Preliminary studies have shown that SMS text messaging and home blood pressure monitoring (HBPM) can be effective in promoting medication adherence and blood pressure control. The best strategy to engage with older patients of low socioeconomic status who are low adopters of technology and disproportionally affected by uncontrolled hypertension is still unknown.

**Objective:**

The objective of this study is to improve blood pressure control in the older, low socioeconomic status population. The study will test two aims: First, we aim to evaluate the feasibility of conducting a randomized controlled trial by using an SMS-based approach among nonadherent, older patients of low socioeconomic status who have uncontrolled hypertension. Feasibility will be assessed in terms of recruitment rates per month (primary outcome); patient acceptability will be evaluated by monitoring retention rates and SMS response rates and using the validated Systems Usability Scale (secondary outcomes). Second, we aim to estimate the effects of the SMS approach on lowering blood pressure and adherence to antihypertensive medications.

**Methods:**

We will recruit 24 patients of low socioeconomic status with uncontrolled hypertension (systolic BP>140 mmHg or diastolic BP>90 mmHg) showing low medication adherence and taking at least two antihypertensives, who have presented to two outpatient clinics of Wake Forest Baptist Health (Winston Salem, North Carolina, USA). Participants will be randomly assigned to either SMS and HBPM (n=12) or usual care and HBPM (n=12) intervention. Clinicians adjusting the patients’ medications will be blinded to the study assignment. Text messages will be sent from a secure platform to assess medication adherence and HBPM on a weekly basis. The content and delivery frequency of the proposed SMS intervention are based on input from three focus groups conducted in Spring 2019. Participants in both study arms will receive education on HBPM and using an HBPM device. We hypothesize that we will successfully recruit 24 participants and the intervention will be acceptable to the participants. It will also improve medication adherence (assessed by question Medication Adherence Questionnaire scores) and blood pressure control.

**Results:**

Our study was funded in July 2020. As of May 2021, we have enrolled 6 participants.

**Conclusions:**

Our findings will help design a larger efficacy trial to advance the field of eHealth delivery systems particularly for older adults of low socioeconomic status. This study addresses a highly significant topic and targets a population of high morbidity and mortality that has been traditionally underrepresented in clinical trials.

**Trial Registration:**

ClinicalTrials.gov NCT03596242; https://clinicaltrials.gov/ct2/show/NCT03596242

**International Registered Report Identifier (IRRID):**

PRR1-10.2196/18984

## Introduction

The successful implementation of the American College of Cardiology/American Heart Association (ACC/AHA) hypertension guidelines based on the SPRINT (Systolic Blood Pressure Intervention Trial) findings will lead to improvements in the health of the American population and reduce the risks posed by heart disease and stroke [1]. Heart disease is the United States’ leading cause of death [1,2]. These guidelines have lowered the blood pressure (BP) thresholds and recommended incorporating home blood pressure monitoring (HBPM). Low patient adherence to antihypertensive medication is the most significant modifiable patient-related barrier to achieving controlled BP [3]. Medication nonadherence contributes to poor BP control that can lead to further cardiovascular complications, including coronary heart disease and heart failure. Furthermore, hospitalization rates are significantly higher among patients with poor medication adherence [4-6]. Objective measures of medication adherence include electronic monitoring of medication administration (eg, Medication Event Monitoring System, prescription records, and dose counts). These measures are expensive, labor intensive, and difficult to incorporate in routine clinic flows. Subjective measures of adherence include physician reports, self-report, and adherence scales. The 8-question Medication Adherence Questionnaire (MAQ) has been well validated to identify adherence among patients with hypertension, and the scores have been shown to correlate well with a range of objective adherence measures [7].

Preliminary studies have shown that SMS text messaging and HBPM can be effective in promoting medication adherence and BP control [8-11]. When HBPM is used in the surveillance of hypertension, it is more cost effective than office visits in the long run, as it reduces the number of unnecessary return visits and antihypertensive drugs administered to chronic users [12]. The best strategy to engage with older, nonadherent patients of low socioeconomic status who are most affected by uncontrolled hypertension is still unknown. The potential of SMS text messaging to engage patients in their own health care has been met with great enthusiasm because of the relatively low cost, transportability, and the widespread use of this technology [8,13]. Internal medicine and nephrology clinics are two outpatient clinics of Wake Forest Baptist Health Winston-Salem, North Carolina, United States. The demographics of older patients visiting those clinics include predominantly African American patients (60%) who are dual Medicare/Medicaid recipients (60%), suggesting that most of them are of low socioeconomic status. Between November 2018 and November 2019, a total of 8170 patients were seen in the two clinics, of which 5889 patients had uncontrolled hypertension (systolic BP [SBP]>130 mmHg or diastolic BP [DBP]>80 mmHg). Among those, 1840 patients with uncontrolled hypertension were adults over the age of 60 years. These numbers are aligned with the literature, with studies reporting hypertension control rates are lower in patients of lower socioeconomic status [2,14]. In 2018, we surveyed a random sample of 50 patients in the outpatient clinic, and 85% of the sample had access to a cellular phone with SMS capability (CL Campos, MD, unpublished data, March 2017). Therefore, developing and evaluating mobile health tools such as SMS to implement clinical guidelines is essential and particularly relevant in settings like our clinic, where the burden of cardiovascular disease and poor adherence to medications is exceptionally high. Using SMS to support this patient population is logical because of its low cost and widespread use [10]. Self‐monitoring has been shown to be pivotal in the management of patients with other chronic diseases such as diabetes [15]. This pilot study aims (1) to evaluate the feasibility of conducting a full-scale randomized controlled trial of a patient-centric, bidirectional SMS with HBPM intervention for older adults of low socioeconomic status with uncontrolled hypertension (BP>130/80 mmHg) who presented at two clinics at Wake Forest Baptist Health, and (2) to explore intervention effects in BP and medication adherence in both groups.

## Methods

### Sample Characteristics

**Figure 1:**
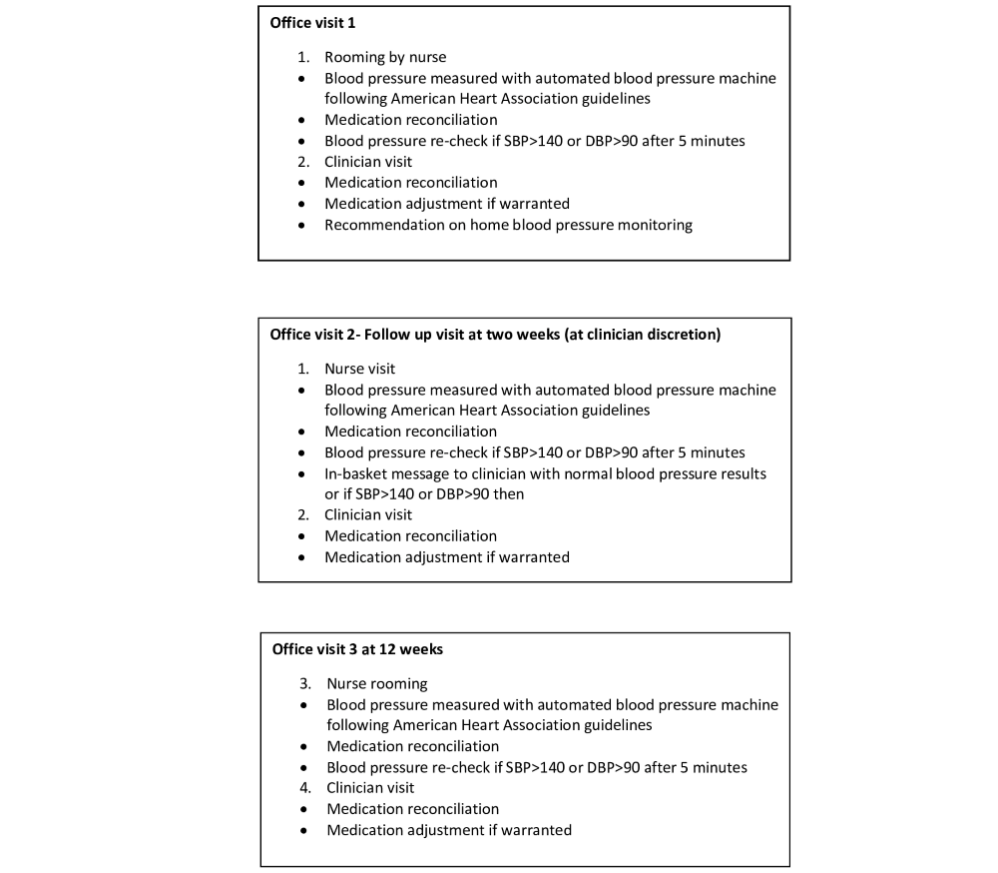
Usual care patient flow. DBP: diastolic blood pressure; SBP: systolic blood pressure.

**Figure 2:**
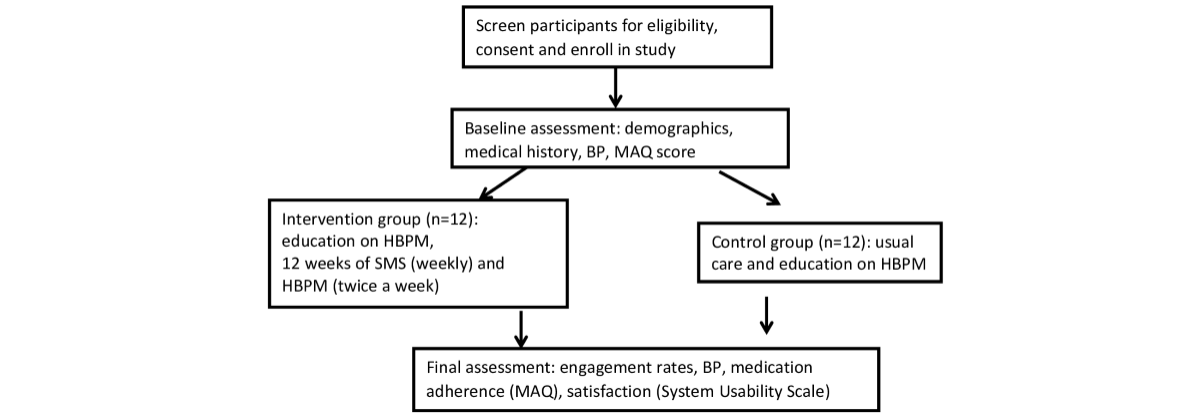
Study algorithm. BP: blood pressure; HBPM: home blood pressure monitoring; MAQ: Medication Adherence Questionnaire.

Twenty-four participants aged 60 years and older will be randomized to either the monitoring intervention or to receive usual care and education; they will be followed-up for 12 weeks. See Figure 1 for patient flow during usual care. The clinicians in the study will identify patients with uncontrolled hypertension during their visits to the clinic. Nurses rooming the patients will record their BP by using an automated BP monitor following the AHA guidelines [16]. The study coordinator will approach prospective patients, that is, those who are 60 years and older, have uncontrolled hypertension (ie, SBP>130 mmHg or DBP>80 mmHg), and have two or more BP medications on their medication list. Nurses will interview the participants in a private room and those with less than college education and with an MAQ score of 0 to 6 will be classified as *nonadherent* and will be invited to participate in the study. The MAQ cut-off score of ≤6 was chosen for this study because it has been used in previous studies and serves a highly sensitive tool for identifying medication nonadherence [17-19]. Low education has been used a proxy for low socioeconomic status [2]. The study coordinator will assess the potential participant’s phone ownership, text messaging ability, text messaging willingness, and receptivity to the intervention (and record all this information). Participants will be excluded if they have end-stage renal disease (on hemodialysis or peritoneal dialysis), a kidney transplant recipient, unable to afford BP medications, institutionalized (hospice or nursing home care) or unable or unwilling to provide consent to participation in this study (ie, dementia or cognitive impairment), or diagnosed with a terminal illness (eg, cancer, chronic respiratory failure, or requiring oxygen support). Participants who are unable to pay for their medications will be excluded as the study will not provide BP medications. The sample size of 12 patients in each group was chosen based on the feasibility and budget [20] (Figure 2).

### Study Intervention

Participants will be randomized to the two study groups by using a computer-based random number generating algorithm. Baseline demographic characteristics of the participants will then be collected. All participants will receive a previously validated Omron Bp785n 10 Series arm blood BP monitor and printed instructions in English or Spanish (as per the AHA) on how to perform HBPM [16]. Participants will be instructed on checking their BP at home at least twice a week [21,22]. Text messages used in the intervention were vetted by patients with hypertension in three focus groups held in the Spring of 2019. A programmer will build the SMS text messaging system to be used for the study intervention by using the Research Electronic Data Capture (REDCap)/Twilio platform, keeping a record of the participants contacted via SMS (sent and received messages). Twilio is a cloud communication platform that embeds messaging directly into REDCap. An example of the SMS system is as follows:

- Adherence checkpoint: *Good morning! Did you take your blood pressure medications today?*
  “No” response: *Let’s try to stay on top of taking them daily! You can do this!*
  Additional prompt: *Did you fill your prescription?*
    If no, alert to study coordinator to place phone call to determine barrier to filling BP medication.
  Additional prompt: *Did you have any side effects from your blood pressure medication?*
    If yes, phone call to determine side effect from BP medication.
  “Yes” response: *Keep it up! [Thumbs up emoji]*
- Self-monitoring phase: *Have you been checking your BP? What is the top number? Bottom number?*

If the participants report SBP below 90 mmHg and or above 180 mmHg and/or DBP below 50 mmHg and/or above 120 mmHg on more than one occasion, those numbers will be considered to be outside of the threshold range. These thresholds are made based on the recommendations by AHA/ACC hypertension guidelines, which designate a hypertensive urgency to be 180/120 mmHg or higher and hypotension urgency to be 90/50 mmHg or lower [23]. The study coordinator will be alerted with any outlier BP recordings, who in turn will alert clinicians through an in-basket message in the electronic medical record (Wake One). The patient will then receive an SMS and a phone call and will be prompted to make a clinic visit.

Patients who do not show up to the follow-up visit will be contacted via phone to assess adherence barriers. The study coordinator will create a telephone encounter in the patient’s electronic health record to document their discussion and route it to the patient’s primary care provider. Patients who refuse participation in the study will be asked for the reason, and their responses will be recorded to determine any barriers to participation. The study coordinator will attempt to contact patients who miss follow-up visits every week up to three times; if the patient still cannot be contacted after three attempts, the patient will be considered “lost to follow-up.” Participants will receive a stipend to compensate them for their time in participating in the study.

### Blinding of Outcomes Assessments

The personnel performing the outcome assessments will be blinded to the participants’ study assignments.

### Statistical Analysis

All statistical analyses will be performed using SAS 9.3 To accomplish Aim 1, we will examine the number of participants enrolled into the study at each study clinic by month over the course of recruitment phase, as well as the average and overall number of participants enrolled per month within each clinic. These rates will assist us in determining the feasibility of recruiting a sufficient sample for the larger trial, and it will potentially inform protocol adjustments. We will estimate additional indicators for feasibility throughout the trial, including measures of retention of participants through the 12 weeks and adherence to the protocol within each group, as well as acceptability of the intervention as measured by the questionnaire (Table 1). We will quantify missing data and dropouts, and we will examine the reasons for ineligibility and discontinuation in the study. For Aim 2, we will examine all data distributions and calculate summary statistics by group using an intention-to-treat approach. Although we will not have adequate statistical power for testing our hypotheses of improved BP control and medication adherence as a result of the intervention, we will conduct an exploratory analysis of any treatment effects (Table 1). An important end product of the trial will be our estimates of variance and correlation for these and other potential outcomes of interest for the larger trial. These estimates will be compared with estimates from studies similar to ours but focusing on other populations and will be used to inform power for the larger trial.

**Table 1:** Evaluation and outcomes.

Outcome and evaluation			Method		Baseline		During the 12-week intervention		At week 12		Expected outcome
**Primary outcome: Feasibility**											
	Recruitment	Track number of enrolled participants per week		✓		✓				24 patients will be recruited in 4 months. The team will learn from the study findings and adjust the protocol to achieve goals.	
**Secondary outcomes**											
	Protocol adherence	SMS response rate/week BP^a^ measurement transmission/week				Weekly				Monitoring of adherence to protocol	
	Acceptability	Systems Usability Scale (SUS) questionnaire. Track participant discontinuation and loss to follow-up Track proportion of patients screened but excluded because they did not own a phone with SMS capability Track participant discontinuation and loss to follow-up Informal comments from participants		✓		Weekly		✓		Monitoring of acceptability; the SUS yields a single score on a scale of 0 to 100. An SUS score >68 would be considered above average.	
**Exploratory outcomes**											
	Systolic BP and diastolic BP change	BP will be measured per clinic protocols and extracted from the patient’s electronic medical record. The average change will be calculated and reported in mmHg.		✓				✓		Systolic BP and diastolic BP will improve	
	Medication adherence	8-item Morisky Medication Adherence Questionnaire (MAQ)		✓				✓		MAQ scores will improve	

^a^BP: blood pressure.

### Data Safety and Monitoring

The proposed study presents small risks to participants. They will receive usual medical care. The principal investigator will be responsible for the overall monitoring of the data and safety of the study participants. We will use REDCap, a secure online platform designed for research, to collect all patient data. Participants’ demographics (including their education level), comorbidities, number of medications, number of BP medications prescribed, and BP measures will be extracted from Wake One records when available. The level of education will be confirmed verbally with each participant during consent process. Only one participant identifier will appear in the data collection forms.

## Results

Our study began recruitment in September 2020, and the anticipated completion date for the recruitment phase is March 31, 2021. This study is funded by CTSI Pilot funding from Wake Forest Baptist Health. This trial has been registered in ClinicalTrials.gov (Identifier: NCT03596242).

## Discussion

The proposed study will evaluate the feasibility of conducting a randomized controlled trial of a new patient-centric SMS delivery system tailored primarily for older adults of low socioeconomic status demonstrating nonadherence to antihypertensive medication. The SMS system was designed with the input of three focus groups composed of older minority patients with hypertension. The study is distinctive in its ability to recruit and test the implementation in a population particularly affected by medication nonadherence and uncontrolled hypertension. To engage patients in health care is considered a key strategy to improve patients' adherence, clinical outcomes, and satisfaction about the care received [24-26]. If successful, a larger efficacy trial will help advance the eHealth delivery system particularly for underrepresented minority patients in the context of BP management. Reducing disparities is a key component of promoting health equity. Assessing interventions aimed to reduce health care disparities are needed to counteract social risk factors in order to achieve health equity [27-29].

We intend to publish the findings of this study. If successful, we will plan to conduct a larger efficacy randomized controlled trial. The dissemination of these results will help improve BP control in this patient population. In addition, our long-term goal is to develop an automated patient-centric system that will improve monitoring of BP and medication adherence in other ambulatory clinics, to help improve BP control rates among older adults with uncontrolled hypertension who visit other primary care clinics at Wake Forest Baptist Health.

## Abbreviations

ACC: American College of Cardiology
AHA: American Heart Association
BP: blood pressure
REDCap: Research Electronic Data Capture
DBP: diastolic blood pressure
HBPM: home blood pressure monitoring
MAQ: Medication Adherence Questionnaire
SBP: systolic blood pressure
SPRINT: Systolic Blood Pressure Intervention Trial
